# Effects of ecological flooding on the temporal and spatial dynamics of carabid beetles (Coleoptera, Carabidae) and springtails (Collembola) in a polder habitat

**DOI:** 10.3897/zookeys.100.1538

**Published:** 2011-05-20

**Authors:** Tanja Lessel, Michael Thomas Marx, Gerhard Eisenbeis

**Affiliations:** Johannes Gutenberg-University, Institute of Zoology/Dep. IV, Soil Zoology and Ecology, Becherweg 13, 55099

**Keywords:** bioindication, community dynamics, drought, flooding, Integrated Rhine Program (IRP)

## Abstract

Within the scope of the Integrated Rhine Program an ecological flood gate and channel was inserted into the polder “Ingelheim” to enhance animal and plant diversity. In 2008, carabid beetles and springtails were collected, using pitfall traps, to measure the effects of ecological flooding and a strong precipitation event at a flood-disturbed and a dry location in this area. At both localities, xerophilic and mesophilic carabid beetle species were dominant throughout the study period. The total number of individuals of hygrophilic species was comparatively constant, while species number increased, partly due to the changed moisture conditions caused by ecological flooding and strong precipitation. Carabid beetle diversity and evenness decreased marginally when ecological flooding was absent. Springtails represent a less mobile arthropod order, and as such the impact of ecological flooding was stronger. An increase in both numbers of species and individuals of hygrophilic and hygrotolerant species occurred in the flood-disturbed location after ecological flooding. After the sites at both locations had dried, the number of individuals belonging to these species declined rapidly. In contrast to carabid species, the strong precipitation event showed no influence on hygrophilic springtail species. Thus, collembolan diversity and evenness decreased markedly in the absence of flooding. We showed that ecological flooding has an influence on the spatial and temporal dynamics of different arthropod groups that inhabit the polder “Ingelheim”. These findings demonstrate the importance of using different arthropod groups as bioindicators in determining the ecological value of a particular polder design.

## Introduction

**Figure 1.**
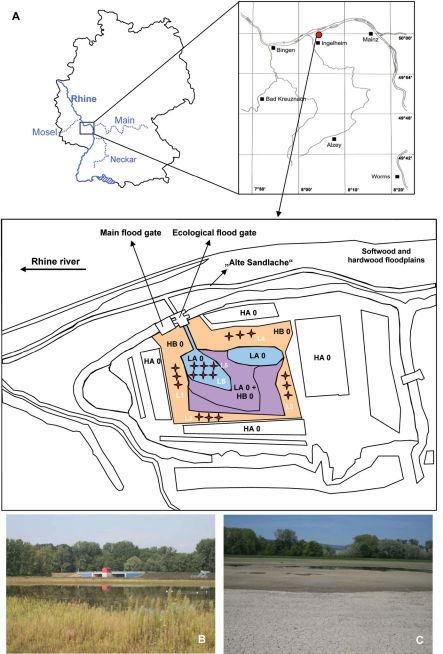
Location of the polder „Ingelheim“ in Germany and location of the different areas and pitfall trap localities (L1–L6) within this polder (**A**). Abbreviations: LA 0: ruderal area; HB 0: fallow area; LA 0 + HB 0: transition area between LA 0 and HB 0; HA 0: agricultural fields; L1–6: locations of the six pitfall trap groups (three pitfall traps per locality). The pictures show the main flood gate (left) and the ecological flood gate (right), and an ecological flooding in March 2007 (**B**) and the fast drying event in the ruderal area after ecological flooding in April 2007 (**C**).

During the last three decades flood protection has become one of the most important goals of countries along the entire course of the river Rhine. Therefore, in 1982 the Integrated Rhine Program (IRP) was established to reduce the economic and ecological impacts of a 200-year flood (an extraordinary flood event, which hypothetically occurs only once in 200 years). The program includes the specific use of hydroelectric power plants, the construction of several polder sites (floodwater retention basins) and the relocation of dikes to enlarge the flooding area of the river Rhine. An essential aim of the IRP is to combine economic (flood protection) and ecological protective measures (Strähle 1992). While it is relatively easy to measure the economic value of such a flood protection area, it is more difficult to evaluate the benefits to plants and animals. The polder “Ingelheim” is an example of a new generation flood prevention site along the Northern Upper Rhine region. It was constructed bearing ecological aspects in mind and completed in September 2006. For the protection of rare plant species found in the ruderal (former seepage) areas (*Isoeto-Nanojuncete*), a smaller gate was inserted (Fig. 1B) in addition to the main flood gate, which is only opened in the case of very high Rhine water levels (water gauge Mainz: > 7.00 m). Only when the main flood gate is opened, the whole polder area (160 ha) is completely flooded. The smaller gate is open most of the time and is only closed after a so-called “ecological flooding” of the smaller ruderal area (20 ha) caused yet by higher Rhine water levels (water gauge Mainz: > 5.00 m). Therefore, ecological flooding of the ruderal area occurs periodically (ca. every eight months) induced not only by higher Rhine water levels but also by the amount of precipitation. In addition to the preservation of the hygrophilic plant community, the aim of ecological flooding is to enhance the diversity and density of animal species and accelerate recovery after flood events. Studies in the polder “Altenheim” showed decreased densities of animal populations after flooding but fast recolonisation after this habitat had dried again (Siepe 1989; 2006).

For this reason the mobile carabid beetles (Coleoptera; Carabidae) and the less mobile springtails (Collembola) were chosen to detect the effects of ecological flooding on these arthropod groups. The ecology and taxonomy of most Middle European species belonging to these two groups have been well researched, making them particularly suitable for such a study. As they can be sampled easily and cost-efficiently, they are also potentially suitable bioindicators (Hopkin 1997; Rainio and Niemela 2003). Carabid beetles are also considered valuable indicators of hydrological conditions in floodplains or other dynamic landscapes (Bonn and Kleinwächter 1999; Ellis et al. 2001; Bonn et al. 2002; Gerisch et al. 2006). In this study the mobile carabid beetles are expected to react relatively quickly to changing moisture conditions, which include abundance and species number shifts between hygrophilic and xerophilic species. Large populations of the less mobile springtails inhabit the soil and are important members of the soil food web (Hopkin 1997). According to Russell et al. (2002) this group is also believed to show very flexible responses to changed habitat conditions and the way they react in flood disturbed habitats is more an adjustment of dominance than the appearance or disappearance of species (Deharveng and Lek 1995; Sterzyńska and Ehrnsberger 1999). Russell et al. (2004) and Russell and Griegel (2006) classified collembolan species of different floodplain habitats into isovalent species groups based on moisture preference. Marx et al. (2009) described several strategies of springtails to survive flooding under both hypoxic and anoxic conditions.

The main aims of this investigation were to determine the effects of ecological flooding on ground beetles and springtails, and to determine their bioindication value. Therefore, results of the 2008 vegetation period are presented, during which both an ecological flood event caused by high Rhine water levels and a flood caused by a strong precipitation event occurred. Between these two flooding events a short but severe drought period occurred at the study site. This vegetation period was of particular importance in answering the main questions posed here because of the fast sequence of the different flood and drought events.

## Material and methods

The Polder “Ingelheim” (49°59'N; 8°03'E, 81–82m a.s.l.) is located in a nature protection area called “Sandlache” near Mainz in the Northern Upper Rhine region. The feed stream of the polder flows through a natural backwater of the river Rhine, the “Alte Sandlache” (Fig. 1A). The central part of the study site was formerly characterised as a ruderal seepage area (now ruderal area) because of seepage water. Ecological flooding, through the ecological flood gate, should prevent the succession of this area from ruderal to fallow. The remainder of the study site is an active agricultural area. After the polder had been built between the agricultural land (HA 0) and the ruderal area (LA 0), an unused fallow area (HB 0) with a dense shrub layer developed (Fig. 1A). This area is dominated by *Limosella aquatica* (L.), *Gnaphalium uliginosum* (L.), *Juncus bufonius* (L.), *Cyperus fuscus* (L.), *Potentilla supina* (L.) and *Lythrum hyssopifolia* (L.) and serves as the riverbank during the ecological flooding of the ruderal area. The ruderal area mainly consists of *Cirsium arvense* (L.), *Conyza canadensis* (L.), *Lactuca serricola* (L.) and *Sinapis arvensis* (L.) and is usually completely flooded during an ecological flood event caused by high Rhine river water levels. During the vegetation period of 2008 the fallow area had a flood disturbance of less than 5% and the ruderal area of more than 30% (flood disturbance was calculated as the percentage of days that sampling could not be performed due to flooding). The soil of the polder is secondary loess with a high sand and loam content, typical of the region. Because of these soil conditions, strong precipitation events are sufficient to flood the ruderal area in particular.

For the study a total of 18 pitfall traps at six locations (three traps per location, distance between the traps: 5m) were used. Two locations were in the ruderal area (L5, L6) and the remaining four locations were situated in the fallow area (L1–L4, Fig. 1A). The pitfall traps had a diameter of 10 cm at ground level and were protected from direct rainwater infiltration by a transparent cover (10 x 10 cm; plexiglas). The traps were filled with a saturated NaCl-solution and detergent as killing agent (Teichmann 1994; Muster 2002). The traps were replaced once every second week and the contents brought to the laboratory, where laboratory ethanol (70%) was used to preserve the catch. The sampling period presented here was from 28 February to 22 October 2008. The ruderal area was flooded from February to May, thus sampling took place for 168 days, while the dry fallow area was sampled for 237 days due to the small number of flood disturbances. The ruderal area dried up very quickly after a flooding event (Fig. 1C). This area was partly flooded again on 25 June 2008 for ca. 20 days as a result of a strong precipitation event (which equated to almost two-thirds of the long-term average of total monthly precipitation). July of 2008 was relatively dry compared to the long-term average of total monthly precipitation (-37.5%). Results are only presented for two localities (L1= fallow area and L6= ruderal area), as it was not possible to determine the collembolan communities in the other four localities (L2–L5) due to the fact that the springtail project ended in May 2008. These two localities are close to each other (< 25 m) but represent different areas and flood disturbances.

Because a number of pitfall traps failed, mainly in the ruderal area (due to flood disturbance), the total number of individuals collected was transformed to the mean number of individuals per trap and day (± Standard error; SE). Diversity (Shannon and Weaver 1949; Wiener 1949) and evenness (Pielou 1975) were first calculated using data from the whole sampling period in order to show the impact of ecological flooding on the different arthropod communities. To determine the influence of the strong precipitation event only, we removed data corresponding to the period of ecological flooding (28 February to 21 May 2008 for the fallow area; 28 February to 18 June for the ruderal area). These indices were calculated to show differences in the community structure between the dry and flood periods. For the comparison of the similarity of carabid beetle and springtail communities of the fallow and ruderal area, the species-based Jaccard and the dominance-based Renkonen indices were used. Furthermore the combined species- and dominance-based Wainstein-index was also calculated to compare the arthropod communities of both locations. This is calculated by the sum of the Jaccard and Renkonen similarities. For carabid beetles, the ecological classification followed Freude et al. (2004) and GAC (2009), and for springtails the classification into isovalent species groups followed Weigmann (1997), Russell et al. (2004) and Russell and Griegel (2006). Dominance classification for both groups followed Engelmann (1978). A PCA was run to show differences in species composition and dominance structure of the two areas. Because of the non-normal distribution of the arthropods in the pitfall traps and the small sample size a non parametric Mann Whitney U-test was calculated to measure significant differences between mean individual numbers caught during the flood and drought events. For statistical analyses Statistica 6.1 (StatSoft company) was used.

## Results

### Carabid beetles

**Figure 2.**
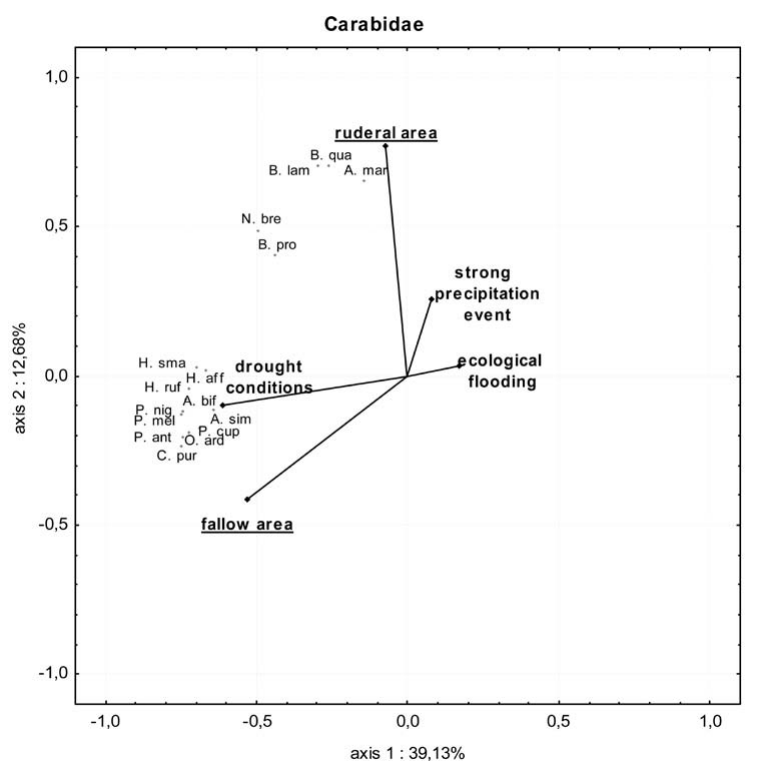
PCA of carabid beetle communities in the fallow area (location 1) and the ruderal area (location 6) during ecological flooding, the flood caused by a strong precipitation event and drought conditions. Only species with more than 1% dominance value in at least one area are included. Abbreviations of the species: **A.mar**=*Agonum marginatum*; **A.bif**=*Amara bifrons*; **A.sim**=*Amara similata*; **B.lam**=*Bembidion lampros*; **B.pro**=*Bembidion properans*; **B.qua**=*Bembidion quadrimaculatum*; **C.pur**=*Carabus purpurascens*; **H.aff**=*Harpalus affinis*; **H.ruf**=*Harpalus rufipes*; **H.sma**=*Harpalus smaragdinus*; **N.bre**=*Nebria brevicollis*; **O.ard**=*Ophonus ardosiacus*; **P.cup**=*Poecilus cupreus*; **P.ant**=*Pterostichus anthracinus*; **P.mel**=*Pterostichus melanarius*; **P.nig**=*Pterostichus nigrita*. Percentage variation explained by the two PCA axes is included.

**Figure 3.**
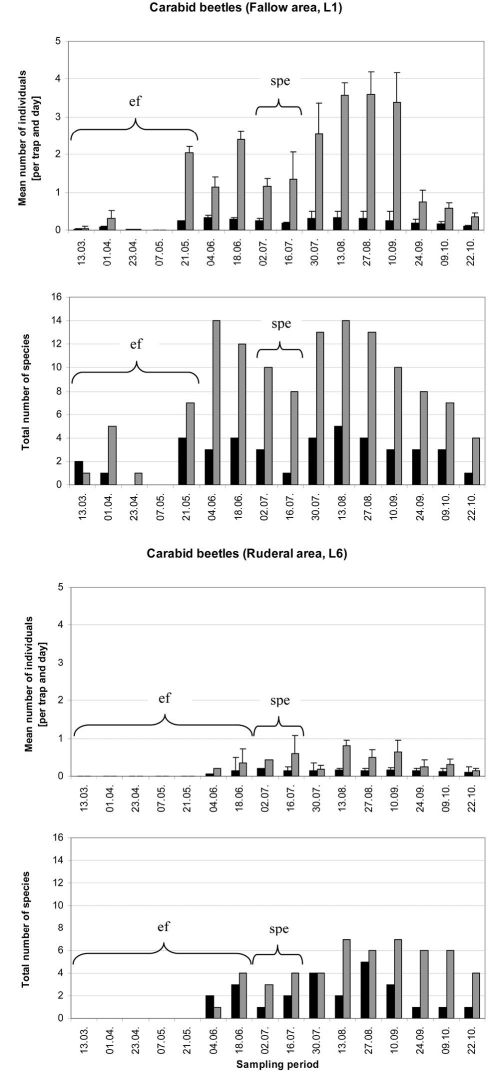
Mean number of individuals per trap and day (± SE) and total carabid beetle species number at location 1 (fallow area) and location 6 (ruderal area) (n=3) during the vegetation period of 2008. Hygrophilic species (black bars) and xerophilic as well as mesophilic species (grey bars) are shown. Abbreviations: ef = ecological flooding; spe = strong precipitation event.

**Figure 4.**
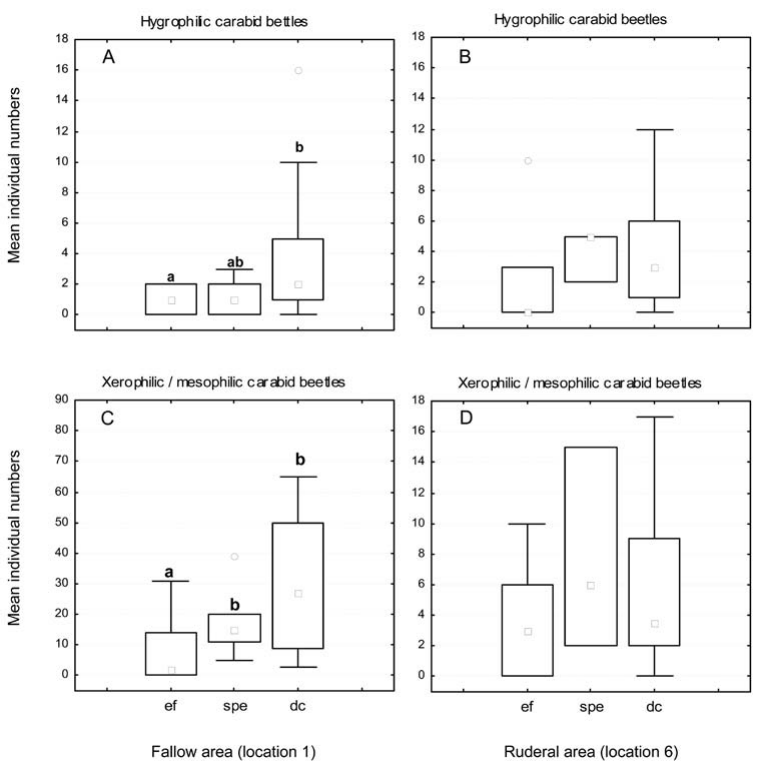
Mean number of individuals of hygrophilic (**A**/**B**) and xerophilic/mesophilic (**C**/**D**) carabid beetle species at the fallow (**A**/**C**) and ruderal area (**B**/**D**) during different moisture conditions. Abbreviations: **ef** = ecological flooding (higher Rhine water levels); **spe** = flood caused by a strong precipitation event; **dc** = drought conditions; ° outliers. Different letters represent statistically significant differences (Mann-Whitney U-test).

In the fallow area, 46 carabid species of 1490 individuals were collected, while 33 species of 514 individuals were collected in the ruderal area. In the fallow area, 26 xerophilic and two mesophilic species dominated, representing more than 64% of all individuals collected, while five eurytopic species comprised more than 28% of the catch. The 12 hygrophilic species only made up 8% of the catch (see Appendix 1). *Harpalus luteicornis* (Duftschmid, 1812) was the only species that could not be clearly classified using the literature and is thus marked uc (unclassified) in Table 1 and in Appendices 1 and 2. In the ruderal area, 12 hygrophilic species dominated the catch (20% of all individuals collected), while 12 xerophilic and one mesophilic species comprised nearly 30% of all individuals. This area was dominated by six eurytopic species, representing almost 50% of the catch, while *Bembidion* species are predominantly limited to the ruderal area (Fig. 2). There were only two species without clear classification (see Appendices 1 and 2). Table 1 shows the classification of the species and individuals with and without the impact of ecological flooding. When the ecological flooding period (and data) was excluded, only a small decrease in abundance and a disappearance of four species were detected in both localities. In the fallow area, the hygrophilic species *Bembidion biguttatum* (Fabricius, 1779), *Ocys harpaloides* (Audinet-Serville, 1821) and *Stenolophus mixtus* (Herbst, 1784) as well as the xerophilic species *Microlestes maurus* (Sturm, 1827) disappeared (Appendix 2). In the ruderal area, in addition to *Demetrias atricapillus* (L.), the hygrophilic species *Anisodactylus binotatus* (Fabricius, 1787), *Bembidion biguttatum* and *Stomis pumicatus* (Panzer, 1796) disappeared (Appendix 2). However, all species that disappeared comprised only small numbers of individuals. This is also confirmed by the comparison of diversity and evenness values with and without the ecological flood data. In both locations without the ecological flood data, Shannon-diversity showed only a small decrease, whereas evenness values remained almost unchanged (Table 1). Furthermore, both areas showed a constant dominance of xerophilic and mesophilic species in terms of species number and abundance over hygrophilic species throughout the vegetation period (Fig. 3). Especially *Pterostichus melanarius* (Illiger, 1798), *Poecilus cupreus* (L.), *Harpalus rufipes* (De Geer, 1774) and *Harpalus affinis* (Schrank, 1781) occurred asdominant and subdominant species (Appendix 2). Thus, ecological flooding appeared not to cause species or dominance shifts. This is also confirmed by the dominance of hygrophilic and xerophilic/mesophilic species during the different moisture periods (Fig. 4). In the fallow area the drought period showed significantly higher abundances of hygrophilic (Fig. 4A: U-test: p ≤ 0.01) as well as xerophilic/mesophilic species (Fig. 4C; U-test: p ≤ 0.01). Higher abundances after the strong precipitation event in the fallow area were only detected for xerophilic/mesophilic species (Fig. 4C; U-test: p = 0.044). In contrast to the fallow area, there were no clear differences between the mean carabid beetle abundances during the flooding and drought periods at the ruderal area (Location 6; Fig. 4B and 4D).

**Table 1.** Total number of carabid beetle and springtail individuals and species at the fallow area (location 1) and the ruderal area (location 6) with and without data from the ecological flooding period. Shannon diversity, maximum diversity and evenness (Pielou) values are also given with and without data from the ecological flooding period. Abbreviations: **e**=eurytopic; **hp**=hygrophilic; **ht**=hygrotolerant; **mp**=mesophilic; **uc**=unclassified; **xp**=xerophilic; **xt**=xerotolerant.

	*Carabid beetles*									*Springtails*								
	Individual number			Species number			Shannon-diversity (Hs)	Maximum diversity (Hmax)	Evenness (Pielou)	Individual number			Species number			Shannon-diversity (Hs)	Maximum diversity (Hmax)	Evenness (Pielou)
	hp	xp, mp	uc, e	hp	xp, mp	uc, e				hp, ht	xt, mp	uc	hp, ht	xt, mp	uc			
*Fallow area*																		
With data of ecological flooding	113	955	422	12	28	6	2.35	3.83	0.61	652	6333	16	3	7	5	1.07	2.71	0.39
Without data of ecological flooding	104	877	411	9	27	6	2.25	3.74	0.60	2	6169	5	1	6	3	0.73	2.30	0.32
*Ruderal area*																		
With data of ecological flooding	101	153	260	12	13	8	2.60	3.50	0.74	3040	2363	2	3	4	2	1.26	2.20	0.57
Without data of ecological flooding	87	140	251	9	13	7	2.54	3.37	0.75	3	2322	1	1	4	1	0.61	1.79	0.34

The different species- (Jaccard) and dominance- (Renkonen) based similarity indices confirmed this stable community structure (Table 2). However, during the entire vegetation period some hygrophilic species with similar numbers of individuals occurred in both areas, although the dominance of the most dominant hygrophilic species varied markedly. In the fallow area, *Pterostichus anthracinus* (Illiger, 1798) and *Pterostichus nigrita* were the most dominant hygrophilic species, whereas in the ruderal area *Nebria brevicollis* and *Agonum marginatum* dominated (Appendix 2). Species with a dominant or subdominant occurrence in only one location were *Carabus purpurascens* Fabricius, 1787, *Pterostichus nigrita* (Paykull, 1790) and *Amara bifrons* (Gyllenhal, 1810) in the fallow area and *Bembidion* *quadrimaculatum* (L. 1761), *Bembidion lampros* (Herbst, 1784), *Nebria brevicollis* (Fabricius, 1792) and *Agonum marginatum* (L.) in the ruderal area (Appendix 2). As such, the species- and dominance-based Wainstein-similarity index values were only about 25%, which is very low given the proximity of the two locations.

**Table 2.** Comparison of the carabid beetle and springtail communities of the fallow area (location 1) and the ruderal area (location 6) using different species based and dominance based similarity indices (Jaccard, Renkonen and Wainstein) with and without data from the ecological flooding period. The percentages show the degree of similarity of the carabid beetle and springtail communities between the fallow and ruderal area. Values higher than 50% represent higher similarity of the communities between the two areas.

*Comparison location 1 with location 6*	*Carabid beetles*			*Springtails*		
	Jaccard	Renkonen	Wainstein	Jaccard	Renkonen	Wainstein
With data of ecological flooding	46.3	52.5	24.3	60.0	53.1	31.7
Without data of ecological flooding	51.1	51.5	26.3	60.0	95.7	57.4

### Springtails

**Figure 5.**
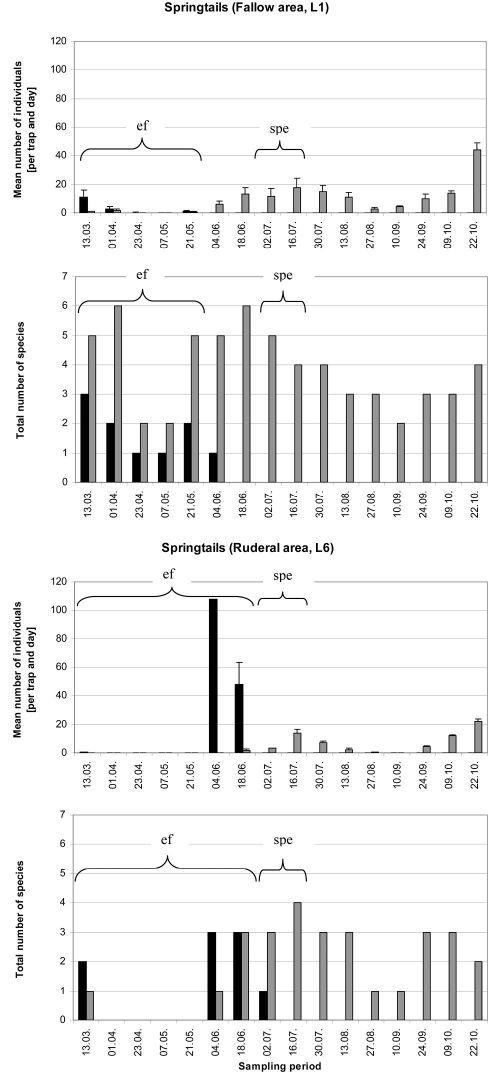
Mean individual numbers per trap and day (± SE) and total species numbers of springtails of the pitfall traps of location 1 and location 6 (n=3) over the vegetation period 2008. Hygrophilic and hygrotolerant species (black bars) and xerotolerant as well as mesophilic species (grey bars) are shown. Abbreviations: **ef** = ecological flooding; **spe** = strong precipitation event.

**Figure 6.**
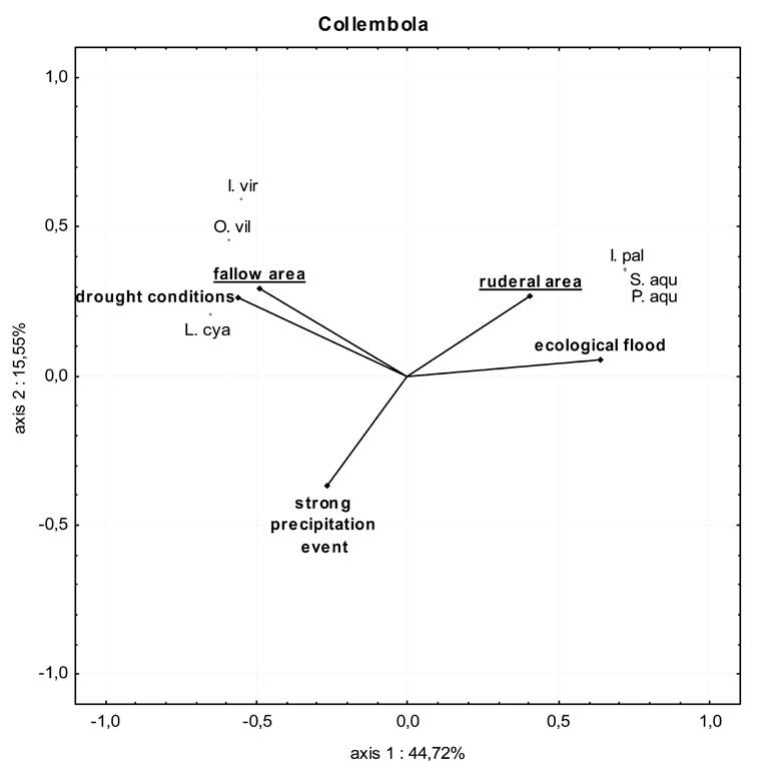
PCA of springtail communities in the fallow area (location 1) and the ruderal area (location 6) during ecological flooding, the flood caused by a strong precipitation event and drought conditions. Only species with more than 1% dominance value in at least one area are included. Abbreviations of the species: **I.pal**=*Isotomurus palustris*; **I.vir**=*Isotoma viridis*; **L.cya**=*Lepidocyrtus cyaneus*; **O.vil**=*Orchesella villosa*; **P.aqu**=*Podura aquatica*; **S.aqu**=*Sminthurides aquaticus*. Percentage variation explained by the two PCA axes are included.

**Figure 7.**
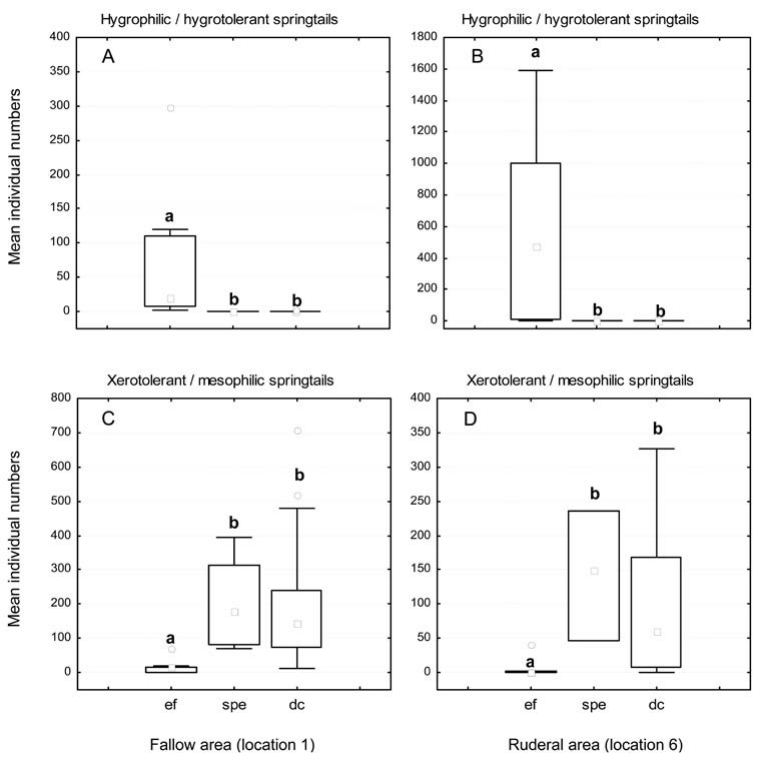
Mean number of individuals of hygrophilic/hygrotolerant (**A**/**B**) and xerotolerant/mesophilic (**C**/**D**) collembolan species at the fallow (**A**/**C**) and ruderal area (**B**/**D**) during different moisture conditions. Abbreviations: **ef** = ecological flooding (higher Rhine water levels); **spe** = flood caused by a strong precipitation event; **dc** = drought conditions; **°** outliers. Different letters represent statistically significant differences (Mann-Whitney U-test).

In the fallow area, 15 collembolan species and 7001 individuals were caught. With the ecological flooding data included, seven xerotolerant and mesophilic species dominated the catch (90% of all individuals collected), while the three hygrophilic and hygrotolerant species comprised less than 10% of all individuals collected (Table 1). Mainly xerotolerant and mesophilic species were detected when data from the ecological flood period were excluded. In the ruderal area, nine collembolan species with 5405 individuals were captured. Here, however, the ecological flooding data showed that three hygrophilic and hygrotolerant species made up 56% of the catch, while four xerotolerant and mesophilic species made up almost 44% of all individuals collected (Fig. 5). Without the ecological flooding data, hygrophilic and hygrotolerant individuals were almost absent, which resulted in a dominance value of almost 100% for xerotolerant and mesophilic species. This result was also reflected in the lower diversity and evenness values (Table 1). In the ruderal area in particular, these indices decreased markedly. During and shortly after ecological flooding caused by a higher Rhine water level, three hygrophilic and hygrotolerant species *Podura aquatica* (L.), *Isotomurus palustris* (Müller, 1776) and *Sminthurides aquaticus* (Bourlet, 1842) were highly abundant compared to all other species in both locations (Figs. 5 and 6, Appendix 3). The mean number of individuals of these species caught in the pitfall traps during the ecological flood was significantly higher than the mean number of individuals caught during the flood caused by the strong precipitation event (Fig. 7A and 7B; U-test: fallow area (L1): p ≤ 0.01; ruderal area (L6): p = 0.025). After the ecological flood event, these species completely disappeared from both areas. Furthermore, compared to the dry period, the strong precipitation event at the end of June had no effect on hygrophilic and hygrotolerant species (U-test: fallow area (L1): p = 0.89; ruderal area (L6): p = 0.36). Compared to the ecological flood event, mean numbers of individuals belonging to the mesophilic species *Isotoma viridis* Bourlet, 1839 and the xerotolerant species *Orchesella villosa* (Geoffroy, 1762) increased significantly during the flood caused by the strong precipitation event and under drought conditions (Fig. 7C and 7D; U-test: fallow area (L1): p ≤ 0.01; ruderal area (L6): p = 0.022). During the sampling period, many collembolan species show a spring and autumn peak with very high individual numbers. In the polder this autumn maximum was also dominated by these two species (*Isotoma viridis* and *Orchesella villosa*). The species-based Jaccard similarity index showed a value of 60% for both areas with and without the impact of ecological flooding, which indicates stable collembolan communities (Table 2). However, differences were obvious concerning the dominance-based Renkonen index and the combined Wainstein index. Without the ecological flooding data, the values of these indices were remarkably high at almost 96% (Renkonen) and 58% (Wainstein), due to the eudominance of *Orchesella villosa* and the dominance of *Isotoma viridis* in both locations (Table 2). However, with the inclusion of the ecological flooding data, these values were lower, mainly because of the influence of the eudominant species *Isotomurus palustris* in the ruderal area. As such, without data from the ecological flooding event, the collembolan communities of both locations were highly similar, while ecological flooding increased the heterogeneity of the collembolan communities of both locations.

## Discussion

The carabid beetle community structure showed clear differences between the two locations. The fallow area is characterized by more vegetation with higher structural diversity and plant heterogeneity, while the ruderal area is characterized by a high level of flood disturbance and less vegetation. The largest number of carabid beetles was collected from the fallow area. The dominance of xerophilic species such as *Harpalus rufipes* or *Harpalus affinis* was expected. A comparatively high number of hygrophilic species were also collected from this area, but with only a small number of individuals. Interestingly, *Agonum marginatum* was found in the fallow area even though this species prefers riverbanks with less vegetation (GAC 2009). This may be an escape strategy of this species to survive extreme flood events (Siepe 1994, Decleer 2003). It was only collected from the fallow area when the ruderal area was flooded. The high dominance of *Pterostichus melanarius* and *Poecilus cupreus* underlines the character of the fallow area. *Carabus purpurascens* is described as a mesophile woodland species and it is possible that this large species prefers habitat in the fallow area where it finds more food and encounters fewer natural enemies (Hildebrandt 1997; Hildebrandt and Handke 1997).

Because of the prolonged flood disturbance from March to May and the strong precipitation event at the end of June 2008 fewer carabid beetles were collected from the ruderal area. Flood events in this area could favour the higher dispersal capacity of pioneer species (Wohlgemuth von Reiche et al. 1999). The appearance of species such as *Amara* *bifrons* or some *Harpalus* species in the ruderal area after flooding confirm the findings of Wohlgemuth von Reiche et al. (1999). Flooding influenced plant species richness as well as carabid species richness. Changing environmental conditions also have a major impact on the presence of carabid beetle species (Brose 2003). Gerisch et al. (2006) underlined the strong relationship between both flood duration and groundwater depth and the occurrence of carabid beetles. The main activity period of carabid beetles is between May and October and the ecological flood event lasted until the end of May. Our result showed that disturbance to the carabid beetle community caused by this flood was very low. The disappearance of only four species, albeit with very low numbers of individuals, confirmed this in particular, because ecological flooding occurred before the onset of the main activity period of many carabid species. After the ruderal area had dried up in July 2008, a migration of eurytopic and xerophilic species, possibly from the fallow area, was observed (*Bembidion lampros*, *Bembidion quadrimaculatum* and *Pterostichus melanarius*). Similar observations were made in a marshland habitat (Decleer 2003).

Furthermore, the dominance of small carabid beetles such as *Bembidion quadrimaculatum* or *Bembidion lampros* can be explained by the work of Schwerk and Szyszko (2007a, b), who found that habitats at an early successional stage are characterised by smaller species compared to habitats of later successional stages. This conclusion was also supported by our results as the less disturbed fallow area included larger carabid beetles (*Carabus purpurascens*). Our conclusion is, thus, that the effect of the 2008 ecological flooding on the carabid beetle fauna was quite minimal.

In contrast to the mobile carabid beetles, ecological flooding had a considerable impact on the collembolan community at both areas. Hygrophilic and hygrotolerant species occurred only during and shortly after this flood event. The adaptation of these species to coping with floods is passive drifting (Coulson et al. 2002; Moore 2002; Hawes et al. 2008). The cuticle of most springtail species is hydrophobic due to its typical structure; it is composed of hexagonal subunits with microtubercles, which varies between different species (Lawrence and Massoud 1973; Eisenbeis and Wichard 1985). Furthermore, the existence of an epicuticular hydrophobic lipid layer was demonstrated by Ghiradella and Radigan (1974). The unwettable properties of the springtail cuticle produces a small air layer (plastron structure), which prevents the species from submersion and enables them to drift passively on the water surface. The feed stream of the polder flows through a natural backwater of the river Rhine, which is colonized mainly by epineustic species such as *Podura aquatica* and *Sminthurides aquaticus* as well as the typical riverbank species *Isotomurus palustris*. This explains both the dominance of these species during and shortly after ecological flooding in both areas and the fact that they quickly disappeared during the short drying period. The fallow area was constantly dominated by xerotolerant and mesophilic species, while the disappearance of hygrophilic and hygrotolerant species caused only a small decrease in diversity and evenness. Hydrological parameters are key factors in determining vegetation structure, carabid beetle and collembolan communities, as well as for other invertebrate taxa (Uetz et al. 1979; Zulka 1993; Russell and Griegel 2006; Siepe 2006; Ilg et al. 2008; Marx 2008). The community structure of the ruderal area showed a higher dominance of hygrophilic and hygrotolerant species; this resulted in a strong decrease of diversity and evenness values when the ecological flood data were excluded. This demonstrates the heterogeneity of the collembolan communities of these two areas, which are probably caused by ecological flooding. The dominance-based similarity indices, in particular, clarify this result. Without the ecological flood data, only the two xerotolerant and mesophilic species *Orchesella villosa* and *Isotoma viridis* dominated both areas. This demonstrates the importance of protecting such rare ruderal areas of this polder with the help of ecological flooding.

## Conclusion

This investigation showed that, in addition to ecological flooding, other flooding events, such as strong precipitation or seepage water, are important factors for the spatial and temporal dynamics of different arthropod groups in such ruderal and seepage areas. These findings emphasize the value of using different taxa in the designing of future polder constructions. If only one arthropod group had been studied this might have led to the erroneous conclusion that ecological flooding has no effect or that it only affects this one bioindicator group. The data collected from several arthropod groups, however, provide more reliable and comprehensive information on the real ecological value of the polder structures.

## Appendix 1.

Dominance [%] of hygrophilic (**h**), xerophilic/mesophilic (**x/m**), eurytopic (**e**) and unclassified (**uc**) carabid beetle species and individuals at location 1 (fallow area) and location 6 (ruderal area):

**Table:**

*Location 1 (fallow area)*	*ec*	*13.03*	*01.04*	*23.04*	*07.05*	*21.05*	*04.06*	*18.06*	*02.07*	*16.07*	*30.07*	*13.08*	*27.08*	*10.09*	*24.09*	*09.10*	*22.10*	
*no. of pitfall traps*		*3*	*3*	*2*	*1*	*2*	*3*	*3*	*3*	*3*	*3*	*3*	*3*	*3*	*3*	*3*	*3*	*total*
*trapping days*		*14*	*19*	*22*	*14*	*14*	*14*	*14*	*14*	*14*	*14*	*14*	*14*	*14*	*14*	*15*	*13*	
*Acupalpus meridianus*	x					2		1										*3*
*Agonum marginatum*	h	3				1	1	1	1									*7*
*Agonum muelleri*	h					1		2				1						*4*
*Agonum sexpunctatum*	h										1							*1*
*Amara aenea*	x						4											*4*
*Amara apricaria*	x													1			1	*2*
*Amara aulica*	x								1		1	1	1	1				*5*
*Amara bifrons*	x						7	16	4		5	2	2	3	3	6	3	*51*
*Amara consularis*	x																1	*1*
*Amara similata*	x						1	2	1	11	8	2	1		1			*27*
*Anchonemus dorsalis*	x							2										*2*
*Badister unipustulatus*	h						1	2			1		1					*5*
*Bembidion biguttatum*	h		1															*1*
*Bembidion lampros*	e								1		1		1					*3*
*Bembidion obtusum*	x	2	9				1											*12*
*Bembidion properans*	e	2			2	2					3							*9*
*Bembidion quadrimaculatum*	e	3				2	3			1	3	1	1			1		*15*
*Brachinus explodens*	x						1											*1*
*Bradycellus harpalinus*	x								1									*1*
*Calathus ambiguus*	x										1	1	1	3	1			*7*
*Calathus fuscipes*	x										1	1	4	1				*7*
*Calathus melanocephalus*	x												1	2	2	2		*7*
*Carabus purpurascens*	m						1	7	9	6	25	64	87	98	18	4		*319*
*Chlaenius nigricornis*	h											1	1	1			1	*4*
*Harpalus affinis*	x					12	6	8	6	5	7	8	2			2	2	*58*
*Harpalus distinguendes*	x		2			1	3					1	1					*8*
*Harpalus latus*	e									1	1		6					*8*
*Harpalus luteicornis*	uc						2					1	1					*4*
*Harpalus rubripes*	x				1		2	1		1	2							*7*
*Harpalus rufipes*	x						1	3	4	10	13	30	16	13	1	1		*92*
*Harpalus smaragdinus*	x					1	3	2	1	2	1	2	2					*14*
*Leistus ferrugineus*	x															1		*1*
*Microlestes maurus*	x		3															*3*
*Microlestes minutulus*	x		1				1											*2*
*Nebria brevicollis*	h					1	2								1			*4*
*Notiophilus palustris*	h											1				1		*2*
*Ocys harpaloides*	h	1																*1*
*Ophonus ardosiacus*	x					1		1	1	2	10	2			1			*18*
*Ophonus azureus*	x					1	1	1			1	1						*5*
*Ophonus laticollis*	x											2	1	1				*4*
*Poecilus cupreus*	x		3			39	16	57	21	19	32	33	32	17	4	10	8	*291*
*Pterostichus anthracinus*	h								2		4	2	3	6	1	3		*21*
*Pterostichus melanarius*	e						9	28	16	7	27	74	94	98	10	17	3	*383*
*Pterostichus nigrita*	h							4	2	2	6	11	9	19	4	5		*62*
*Pterostichus oblongopunctatus*	m													3				*3*
*Stenolophus mixtus*	h					1												*1*

## Appendix 2.

Total number of carabid beetle individuals (Carabidae), number of flood-undisturbed pitfall traps and trapping days of location 1 (fallow area) and location 6 (ruderal area). Abbreviations: **ec**=ecological classification, **h**=hygrophilic, **x**=xerophilic, **m**=mesophilic, **uc**=unclassified, **e**=eurytopic.

**Table:**

*Location 1*	*ec*	*13.03*	*01.04*	*23.04*	*07.05*	*21.05*	*04.06*	*18.06*	*02.07*	*16.07*	*30.07*	*13.08*	*27.08*	*10.09*	*24.09*	*09.10*	*22.10*	
*no. of pitfall traps*		*3*	*3*	*2*	*1*	*2*	*3*	*3*	*3*	*3*	*3*	*3*	*3*	*3*	*3*	*3*	*3*	*total*
*trapping days*		*14*	*19*	*22*	*14*	*14*	*14*	*14*	*14*	*14*	*14*	*14*	*14*	*14*	*14*	*15*	*13*	
*Ceratophysella bengtssoni*	uc	3	4		1			1	1									*10*
*Ceratophysella denticulata*	uc		1															*1*
*Neanura muscorum*	m		1															*1*
*Podura aquatica*	h	13	50			12												*75*
*Isotomurus palustris*	h	437	106	10	4	15	2											*574*
*Isotoma viridis*	m	34	24	1	1	1	59	101	56	56	47	32	8	2	8	125	753	*1308*
*Entomobrya lanuginosa*	m	2	41			2	5	1										*51*
*Lepidocyrtus cyaneus*	x	3	4			2	27	32	42	33	23	6	1		7	39	77	*296*
*Lepidocyrtus paradoxus*	uc		1															*1*
*Orchesella villosa*	x	9	5	2		15	162	411	377	638	543	429	106	192	412	453	876	*4630*
*Bourletiella hortensis*	uc							2										*2*
*Sminthurus viridis*	x		11			3	2	10	1								1	*28*
*Sminthurus nigromaculatus*	m	2			1			6	7	2	1							*19*
*Sminthurides aquaticus*	h	3																*3*
*Deuterosminthurus pallipes*	uc				1		1											*2*

## Appendix 3

Total number of springtail individuals (Collembola), number of flood-undisturbed pitfall traps and trapping days of location 1 (fallow area) and location 6 (ruderal area). Abbreviations: **ec**=ecological classification, **h**=hygrophilic/hygrotolerant, **x**=xerotolerant, **m**=mesophilic, **uc**=unclassified.

**Table:**

*Location 6 (ruderal area)*	*ec*	*13.03*	*01.04*	*23.04*	*07.05*	*21.05*	*04.06*	*18.06*	*02.07*	*16.07*	*30.07*	*13.08*	*27.08*	*10.09*	*24.09*	*09.10*	*22.10*	
*no. of pitfall traps*		*2*	*-*	*-*	*-*	*-*	*1*	*2*	*1*	*2*	*2*	*3*	*3*	*3*	*3*	*3*	*3*	*total*
*trapping days*		*14*	*-*	*-*	*-*	*-*	*14*	*14*	*14*	*14*	*14*	*14*	*14*	*14*	*14*	*15*	*13*	
*Ceratophysella bengtssoni*	uc	1																*1*
*Podura aquatica*	h						192	65	67									*324*
*Isotomurus palustris*	h	7					1318	823	388	3								*2539*
*Isotoma viridis*	m	3					1	5	4	66	19	6			98	188	177	*567*
*Lepidocyrtus cyaneus*	x							2	1	9	1	3			2	1		*19*
*Orchesella villosa*	x							34	41	308	178	90	15	7	79	349	675	*1776*
*Sminthurus viridis*	x									1								*1*
*Sminthurides aquaticus*	h	9					75	76	17									*177*
*Deuterosminthurus pallipes*	uc												1					*1*
